# Draft Genome Sequence of the First Confirmed Isolate of Multidrug-Resistant *Mycobacterium tuberculosis* in Tasmania

**DOI:** 10.1128/genomeA.01230-17

**Published:** 2017-11-02

**Authors:** Sanjay S. Gautam, Micheál Mac Aogáin, Ronan F. O’Toole

**Affiliations:** a School of Medicine, Faculty of Health, University of Tasmania, Hobart, Tasmania, Australia; b Department of Clinical Microbiology, School of Medicine, Trinity College Dublin, St. James’s Hospital, Dublin, Ireland

## Abstract

The spread of multidrug-resistant (MDR) tuberculosis (TB) has become a major global challenge. In 2016, Tasmania recorded its first known incidence of MDR-TB. Here, we report the draft whole-genome sequence of the *Mycobacterium tuberculosis* isolate from this case, TASMDR1, and describe single-nucleotide polymorphisms associated with its drug resistance.

## GENOME ANNOUNCEMENT

The earliest written record of tuberculosis (TB) in Tasmania comes from Colonel David Collins, who reported in 1804 that a member of his Hobart settlement had consumption (1). Today, Tasmania is regarded as a low-TB-burden state with an incidence rate of 1.7/100,000 persons compared to 5.7/100,000 nationally in Australia in 2014 (2). Until recently, Tasmania had been free of multidrug-resistant (MDR) forms of TB; however, in 2016 its first case of MDR-TB was reported.

We have previously characterized the genomes of MDR and extensively drug-resistant (XDR) *Mycobacterium tuberculosis* (3–5). Here, genomic DNA of the Tasmanian isolate, TASMDR1, was sequenced using an Illumina MiSeq instrument. A total of 2,860,297 paired-end reads were mapped to the publicly available annotated genome of *M. tuberculosis* reference strain H37Rv (GenBank accession number NC_000962.3) (6) by Burrows-Wheeler alignment (7). This yielded an average read depth of 65.5-fold, covering 97.8% of the H37Rv genome. Variants relative to the H37Rv reference genome were called using the SAMtools analysis suite, and variant annotation was performed using SnpEff (8, 9). A 4,230,496-bp draft genome assembly of 220 contigs was assembled *de novo* using the SPAdes assembler (v3.7) (10). Assembled contigs were ordered with respect to the *M. tuberculosis* H37Rv genome using ABACAS (11).

A total of 1,553 variant sites were identified relative to the H37Rv genome and consisted of 1,408 single-nucleotide variants (SNVs) and 145 insertions/deletions. Of the variants, 881 were nonsynonymous; of these, 784 were SNVs and 97 were insertions/deletions. The genome of TASMDR1 displayed high-confidence single-nucleotide polymorphisms in genes correlating with antimicrobial drug resistance when analyzed using the PhyResSE database (12). These include high-confidence mutations in the *katG* gene (aGc/aCc, S315T) and *rpoB* gene (gAc/gGc, D435G; tCg/tTg, S450L), which underlie *M. tuberculosis* resistance to isoniazid and rifampin, respectively (13, 14). These data establish the genetic bases of the MDR phenotype exhibited by strain TASMDR1.

Additional mutations were detected in the *embB* gene (Atg/Gtg, M306V) and *pncA* gene (cCg/cTg, P62L) that are associated with resistance to ethambutol and pyrazinamide, respectively (15–18). Furthermore, an A/C substitution was detected at position 514 of the 16S rRNA gene, *rrs* (MTB000019), and is related to streptomycin resistance (19, 20). The TASMDR1 isolate belongs to the Beijing sublineage of East Asian Lineage 2, as predicted by the PhyResSE and TB Profiler databases (12, 21).

The drug-resistance mutations that were identified in the genome of the TASMDR1 isolate were detected within a significantly shorter turn-around time compared to conventional phenotypic drug-susceptibility testing. Although MDR-TB isolates are currently rare in Tasmania, this study highlights the utility of having a microbial whole-genome sequencing facility available for rapidly determining the resistance profiles of MDR-TB isolates that may present in a low-TB-incidence setting.

### Accession number(s).

This whole-genome shotgun project has been deposited at DDBJ/ENA/GenBank under the accession number NTFG00000000. The version described in this paper is version NTFG01000000.
